# Localized Delivery of Drugs through Medical Textiles for Treatment of Burns: A Perspective Approach

**DOI:** 10.34172/apb.2021.030

**Published:** 2020-07-15

**Authors:** Ruchi Tiwari, Gaurav Tiwari, Akanksha Lahiri, Vadivelan R, Awani K Rai

**Affiliations:** ^1^Department of Pharmacy, Pranveer Singh Institute of Technology, Kalpi Road, Bhauti, Kanpur-208020, India.; ^2^Department of Pharmacology, JSS College of Pharmacy, Ooty-643001, India.

**Keywords:** Burn wound, Nanofibers, Methods to manufacture nanofibers, Applications of nanofiber

## Abstract

The topical delivery offers numerous benefits, such as the ability to deliver drugs specifically on site selectively, prevents fluctuations in the levels of the drug, improved compliance, and improved self-medication capacity. Skin is the main route of the administration of the drug delivery system (DDS) and burns mainly cause skin damage. A burn is a kind of damage caused to skin and tissues by fire, ice, electrical energy, pollutants, friction, and radiation. There are three different types of burns, including superficial epidermis burns, partial-thickness dermis that stretch to the papillary and reticular dermis, and full-thickness burns that cover the dermis whole. The objective of the present review article is to focus on fabrication techniques of medical textiles, different types of polymers used for designing medicated textiles, skin burn conditions, and application of medicated textiles for treatment of burn along with other applications. Cream, ointment, and gel are the dosage forms used in burns. Intravenous fluids, wound care, assorted antibiotics, surgical and alternative medicines, burned creams and salami, dressings can be used to treat wounds. Nanofibers are nanometer-specific fibers that encapsulate drugs inside them and cure wounds. Nanofibers have all the properties that speed up wound healing. The properties are mechanical integrity, proper timing of wound addiction, temperature homeostasis facilitation and gas exchange, absorption of exudates. The nanofibers have been used in burn care and have been highly efficient and non-toxic.

## Introduction

The importance of the topical distribution of drugs is growing. The method of topical distribution of pharmaceutical substances is delivered everywhere in the body as topical routes by face, rectum, vaginal, and skin.^1^ Skin is one of the organs on the human body most readily accessible, which makes the perfect location to topically administer drugs. Skin is also the primary way to deliver topical medications. The presence of skin surface lipids can have a significant impact on topical medications.^2^ The supply of medicaments from topical local or systemic formulations essentially involves passive skin diffusion.^3,4^ The ability to deliver specific drugs on-site selectively and avoid fluctuations in drug level, improving patient compliance, and improving the capacity for self-medication offer many advantages. While the skin is the biggest body organ, it is less valuable as well. This acts as a protective barrier protecting the outer layer of the human body to prevent injury, heat, malnutrition, and disease of inner tissues.^5^ It also aids the body’s thermoregulation process by the action and vasodilatation of hair follicles. The skin collects sensory information from the environment, supports vitamin D synthesis and plays a decisive role for the system’s activation against external pathogens. It consists of the epidermis, dermis, and hypodermis, which form three main primary levels.^6^ The wide skin surface (near 20 square feet) makes it a potential way to deliver drugs. Topical drug distribution is primarily intended to be local, where (i) the need for systemically distributed drugs can likely be avoided, (ii) the maximum dosage needed to reach the target location (skin) may be decreased, and (iii) adverse effects at target site reduced. Several topical dermatological services contain skin anti-fungal therapies, sunscreen, keratolytic therapy, local analgesic, disinfectant, and skin disorders like psoriasis.^7^ Apart from it, the transdermal medicament distribution was meant for a systemic drug impact where the skin is just the entrance to the body for the drugs. Transdermal patches are for instance used to provide smoking cessation nicotine, chronic pain buprenorphine, and opioid and activity condition hyoscine (also known as scopolamine). Penetration of drugs across SC is governed by the second law of Fick’s for both topical and transdermal drug supplies.^8,9^ The medication may stay nearby or cross into a dermis relying upon the properties and technique for delivery where, if hydrophilic, it tends to be ingested rapidly through vessels into the circulation.^5,7^ The fine capillaries stretch into the upper layers of the dermis directly underneath the dermato-epidermal intersection. Shunts made by hair follicles facilitate small molecules of hydrophilic or hydrophobic drugs to penetrate across SC^10^ while the delivery of large molecules like siRNA, DNA, peptides, and enzymes consistently been considered as basic research classification because their molecular size is >500 Dalton (Da). The Nanotechnology approach suggested by researchers increases the concentration as well as the flow of medicines in the blood circulation.^11^ The distribution of hydrophobic and hydrophilic drugs can be achieved through both nanoparticles and nanofibers and can control its release over a long period time. Together, the diagnosis of many common dermatological disorders and large market sales have significantly affected these systems.^12^

## Skin and burn 

Skin is nearly exposed to the outside world and thus susceptible to continuous wear and injury. Burn injuries are some of the most frequent among skin injuries that require medical attention.^13^ Burning is a form of skin and tissue injury caused by fire, cold, energy, chemical substances, waste, and radiation.^7^ The warmth from hot liquids, solids, and flame is usually the cause of burns.^14^ The rate of burn in women and men is nearly similar but the causes may be different.^15^ Burning can be caused in women through open cooking fires or unsafe cookery stoves. Burn in men can be associated with the workplace. Three kinds of burns occur, namely superficial burn, partial burn thickness, full burn.^16,17^ Their type, layers, appearance, texture, feeling, cure time, predictions, and examples given in Table 1.^18-21^ Intravenous liquids, trauma care, drugs, surgery, and complementary therapies can be used to control burns. In burns, the dosage forms used are cream, ointment, and gel. Burning can affect some or all of the layers of the skin and is triggered by hot solids, hot fluids (scalding), or fire (Source: International Society for Burning Injuries). Nevertheless, burn injuries are also known as induced by cigarette burns related to fire, radioactivity, ultraviolet, chemicals, and breathing effects.^22^

**Table 1:** Table depicting different types of burn with details^18-21^

**Type**	**Layers involved**	**Appearance**	**Texture**	**Sensation**	**Healing Time**	**Prognosis**	**Example**
Superficial (first-degree)	Epidermis	Red without blisters	Dry	Painful	5-10 day	Heals well. Repeated sun burns increase the risk of skin cancer later in life.	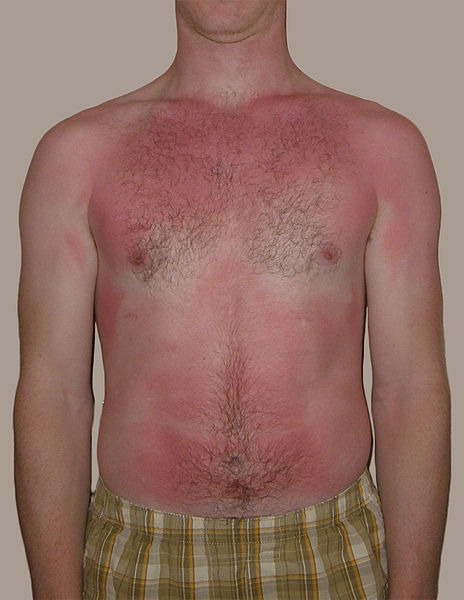
Superficial partial thickness (second-degree)	Extends into superficial (papillary) dermis	Redness with clear blister. Blanches with pressure	Moist	Very painful	2–3 week	Local infection (cellulitis) but no scarring typically	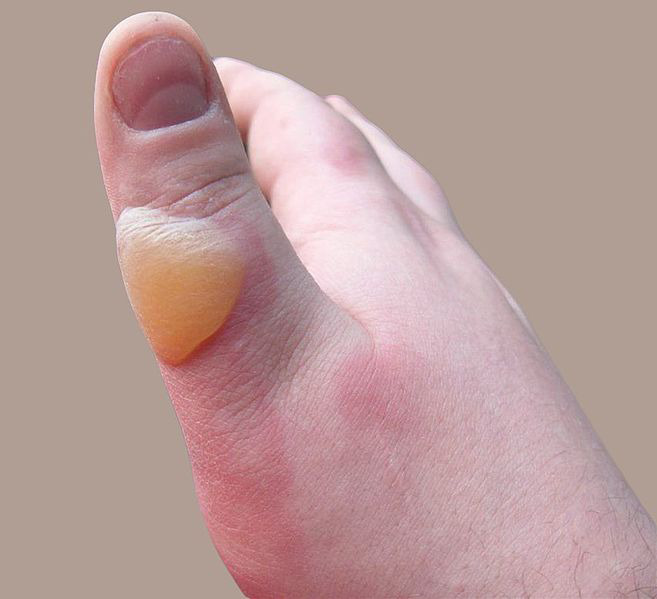
Deep partial thickness (second-degree)	Extends into deep (reticular) dermis	Yellow or white. Less blanching. May be blistering	Fairly dry	Pressure and discomfort	3–8 weeks	Scarring, contractures (may require excision and skin grafting)	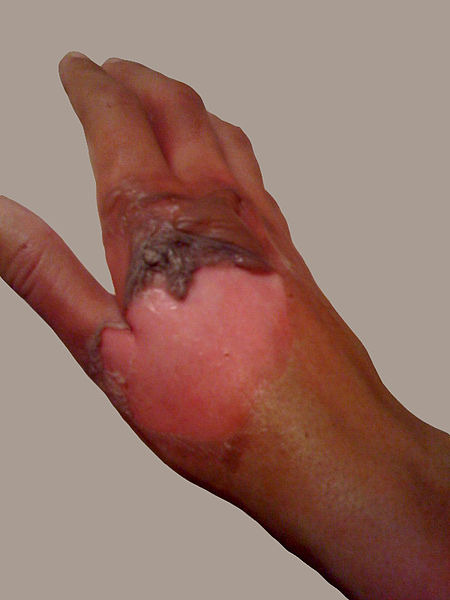
Full thickness (third-degree)	Extends through entire dermis	Stiff and white/brown. No blanching	Leathery	Painless	Prolonged (months) and incomplete	Scarring, contractures, amputation (early excision recommended)	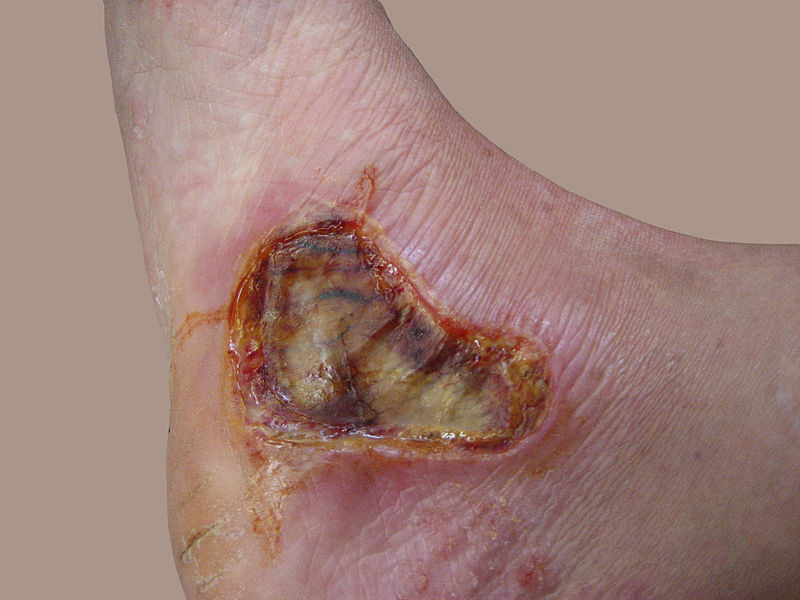
Fourth-degree	Extends through entire skin, and into underlying fat, muscle and bone	Black; charred with a scar	Dry	Painless	Requires excision	Amputation, significant functional impairment and in some cases, death.	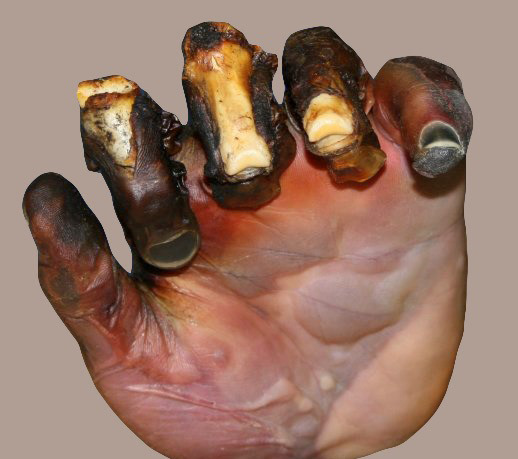

Wound dressings could be an effective approach in avoiding bacterial infections in burning apart from washing wounds and using different topical anti-microbial agents. The adequacy of a wound fabric depends on the type of fire. Conventional dressings are not adequately active to induce haemostasis, bind, and sustain a damp environment around the cut. As an outcome of the advancements in the field of nanotechnology, it is presently possible to plan nanofiber dressings (NFDs), where a nanofibrous covering is added to a fundamental material supporting the surface.^23^ NFDs have preferred over other topical and transdermal systems because of the following reasons: Nanofibers in the extracellular matrix (ECM) are believed to imitate collagen fibrils.^17^ These attributes of nanoparticles not only boost their tissue permeation and retention effect but also increase therapeutic efficacy, contributing to lower toxicity of human cells or tissues.^18,14^ Nanofibers can form highly porous meshes, reveal high surface area, strong cell adherence, regulated *in vivo* biodegradation rate, and thus used for biomedical applications and wound treatment.

In other aspects, NFDs work in a humid environment and need not change frequently, thus minimizing discomfort and bruises which are very useful for survivors of burns.^24^

## Burn wound healing progression

**Figure 1:**
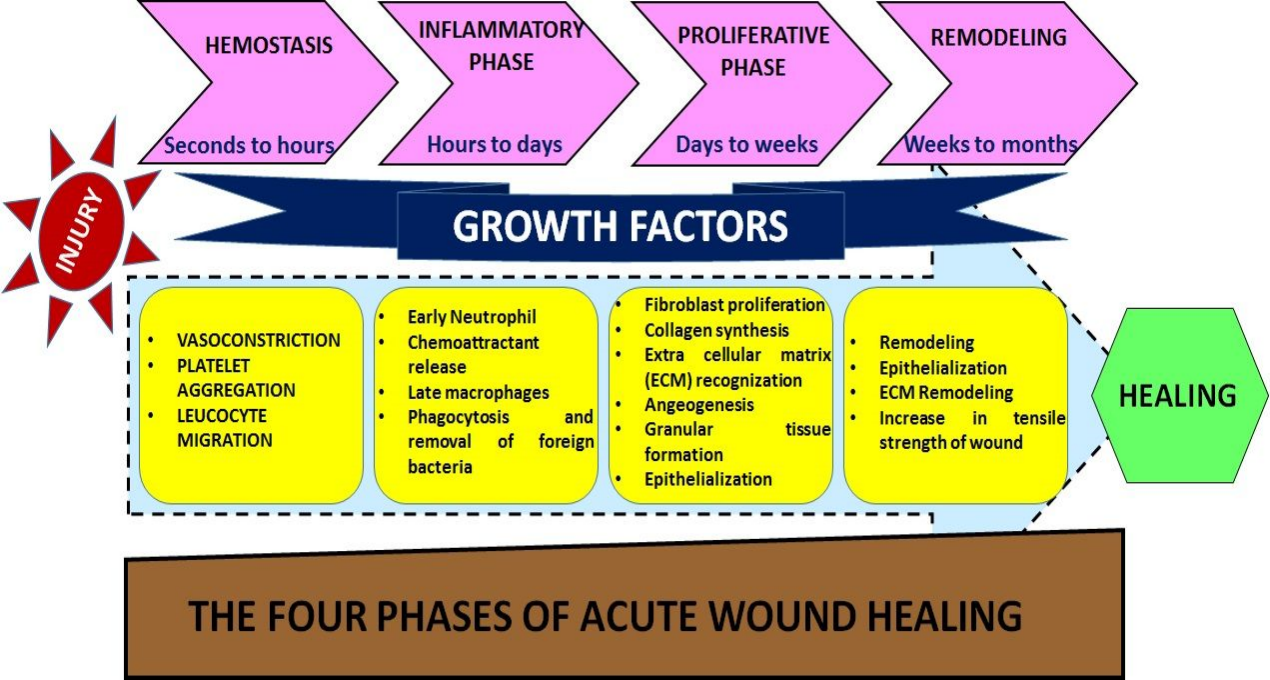
Healing of wound produced from burn

The development of burn wounds is persistent tissue necrosis in the region of stasis after the initial thermal injury has been through. Several chemical and mechanical factors contribute to the local pathophysiological pathways for the treatment of burn wounds.^25^ Fast inflammation contributes to cytokines and free radicals aggregation together with a neutrophil plugging in dermal venules. Enhancing vascular permeability and changes in hydrostatic interstitial tension contribute to edema with vascular obstruction.^26^ By thrombosis, hypercoagulability also impairs blood flow, whereas oxidative stress kills endothelial cells and threaten vascular pathology.^27,28^ The localized areas of a burn wound are three: the main coagulation area, the intermediate stasis region, and the hyperemia outer zone.^12^ The skin and the tissues below the surface repair injury^13^ in the following complex process. Blood clotting, swelling, tissue growth, and tissue reshaping are repetitive phases of repair. Among different stages,^14^ blood coagulation is considered as inflammation cycle (Figure 1).

### Hemostasis 

After the injury, platelets aggregate at the injured site and trigger many events. Platelets trigger the release of fibrin and promote clotting which slows down or prevents bleeding, and tends to protect the injured vessels.^15^ Cell proliferation is mediated by platelet-derived growth factors released at injured site.^29^

### Inflammation 

This phase consists of the removal of dead and damaged cells along with bacteria and pathogens from the site of injury by white blood cells during the phagocytosis process.

### Proliferation 

Angiogenesis, secretion of collagen, development of granulation, epithelialization, and wound closure take place at this stage.^16^ In angiogenesis, new blood vessels develop vascular endothelial cells.^17^ Fibroblasts are formed in fibroplasia and tissue granulation and by excreting collagen and fibronectin they are forming a new temporary ECM. At the same time, the epidermis re-epithelialization occurs, with the proliferation and “crushing” of the epidermis over the wounded bed providing cover for the new tissue.^30^

### Maturation (Remodelling) 

This phase involves rearrangement of collagen at the injured site and dead or damaged cells are removed by apoptosis.^31^

## Wound healing approaches associated with nanofibers

Dressings are bioactive goods that provide active ingredients for wound healing, whether through the use of bioactive composites or from materials with endogenous activity.^32^ Bioactive dressing products are designed to deliver active compounds in the care of wounds. Most of the commercially available bioactive dressings are based on the occlusion theory for wounds, typically through the reduction of scab development by penetration of the skin exudate secreted from the ulcer. Such dressings secure the injury in a moist environment to avoid moisture loss and dehydration.^33^

This consists of polymers that are processed in a particular way to form threads of several micrometers through nanometers. The large area of volume and the handling of surface properties allow nanofibers to shape superfine structures in perfect matrix.^34^ The scaffolds are typically used for organic uses because of their high porosity and large volume to the surface.^18^ It is important to use all natural and synthetic polymers. The development of new wound-healing technologies through nanofiber technology can be improved dramatically. Nanofiber scaffolds provide wound dressing qualities like mechanical integrity, suitable adherence to an injury, the non-occlusive capacity to encourage temperature homeostasis, permit gas exchange, and absorb exudates.^35^

## Naturally occurring polymers

Natural materials have received new publicity due to their high biocompatibility and environmentally friendly characteristics for biomaterial applications.^36^ Natural polymers are commonly used in wound dressings because they are simulated with ECM and thus biologically acceptable and inhibit immunological reactions that are observed in synthetic polymers.^37^ Natural polymers are typically followed by synthetic polymers because of their inherent biocompatibility properties and bioabsorbable materials.^38^ There are no ecological or economic problems and they are not generally harmless even at high concentrations to the human body.^25,26^

### Polysaccharides 

Glycans are a group of natural polymers made up of monosaccharide and its derivatives.^27^ Polysaccharides that contain only one monosaccharide form are known as homopolysaccharides or homoglycans, whereas heteropolysaccharides or heteroglycans are known to possess more than one monosaccharide.^28^ Strong hemocompatibility and low costs are advantages of the heparin-like structure of polysaccharides.^29^

### Cellulose

Cellulose is the natural polysaccharide developed most abundantly by bacteria or plants and a positive homopolysaccharide generated from residues of D-glucopyranose connected by glycosidic linkages of β-(1, 4).^30^ It has also a fibrous structure similar to a fibre network of 3-40 nm in diameter and 1-9 μm in size.^31,32^ With an emphasis on poorly soluble products, Löbmann and Svagan illustrated the usage of cellulose nanofibers in product formulations.^39^ Sheikhi et al hypothesized on the advent of novel nanocellulose forms, including hairy cellulose nanocrystals bearing crystalline (resistant to biodegradation) and amorphous (ready to biodegrade) cellulosic regions for flexible cargo distribution with precision medicine applications.^40^

### Chitin

Chitin is the second-largest polysaccharide and is the structural component of fungal and yeast fungi or cell walls of arthropods.^35^ Chitin is a fundamental antimicrobial homopolysaccharide composed of β-(1, 4), the glucoside-referenced residue of N-acetyl D, depending on the source of the nitric album and appears in two α- and β groups. Chitin is used in the processing of another homopolysaccharide called chitosan industrially through a method of thermochemical deacetylation.^36^ By modifying the esterification and by nanofibrillating ultrasound, Wang et al developed Chitin nanofibers with a diameter of around 15 nm from easily available chitin material. Ultrasound therapy also provided an increase in crystallinity to the chitin nanofibers (from 57.31 to 74.25 percent).^41^

### Chitosan

Chitosan may be used as an antimicrobial agent in terms of its immediate effect on some fungi, algae, and bacteria.^35^ There were two suggested pathways to explain chitosan antimicrobial activity (see Drury and Mooney for analysis).^36^ The first mechanism is due to the polycationic nature of chitosan which affects and increments its permeability with negatively charged residues on cell membranes. The second process is deoxyribonucleic (DNA) binding with chitosan which interferes with the production of ribonucleic acid (RNA).^37^ The cotton cellulosic film with thin layers of chitosan and hyaluronic acid (negative charged) was immobilized by Arslan et al.^38^ Chitosan can facilitate wound healing by stimulation of fibroblast, the deposition and organization of collagens, cell migration, and the granulation, and vascularisation of stimulants.^39^ Karimi et al utilized chitosan, polyethylene oxide, cysteine and drugs respectively to fabricate non-woven mucoadhesive fibre mat by electrospinning process. Scientists reported that high concentrations of vancomycin were released in the first 24 hours through biodegradable mucoadhesive nanofibrous membranes, however, the amphotericin-B release was influenced by more regulated phenomena.^42^

### Hyaluronic acid 

Hyaluronic acid effects on the cellular processes, including the healing of injuries, in all living organisms that communicate with cell surface receivers. For pediatric purpura cases even hyaluronic acid dressings were used effectively to treat skin lesions.^41^ Ahire et al prepared nanofibers of hyaluronic acid/poly(D, L-lactide) (HA/PDLLA) and studies revealed that the release of HA from HA/ PDLLA fibres was at a regular and steady state.^43^

### Carrageenan

Hydrocolloids are classified as carrageenans and are derived from the sea plant group Rhodophyceae. Carrageenans consist of several integrated systems in contrast to the alginate system. Carrageenans are available commercially as a blend of three carrageenan styles. The ionotropic and cold-set pathways were active in the gel environment of carrageenan. Production of cellulose nanofibers (CNF) from agricultural waste was demonstrated by Johnson et al and further CNF were modified with the use of oligosaccharides (CO) for drug delivery.

However, researchers concentrated on the antimicrobial action of CO-CNF charged by surfactin against periodontal pathogens. It has been observed that CO-CNF which is filled with surfactin has possible antimicrobial action against periodontal pathogens.^44,45^

### Alginate

Alginates, derived from dried algae, are unbranched polysaccharides used as wound dressings. Alginates are absorbent and form hydrophilic gels, creating a wet wound-healing atmosphere.^46^ The main reason for using alginates in wound dressings is their haemostatic potential. The coagulation effects of zinc and calcium alginate dressings have been contrasted to non-alginate dressings. It was observed that alginate dressings were more effective when compared to non-alginate dressings in this way. Zinc containing alginates had the highest haemostatic potential.^47,48^ An ion-exchange reaction occurs when an alginate dressing comes into contact with a flushing cut. Alginate dressing calcium ions are substituted for skin fluid ions or wound exudates and the dressing swells with sodium ions.^49^

### Pectin 

Pectin is another organic macromolecule used along with cellulose and artificial polymers that is part of many commercially available dressings. Pectin may function as a barrier to the production of bacteria in the acidic environment.^50^

### Fucoidan 

Fucoidan is a sulphated polysaccharide. Because of its anticoagulant ability, close to that of heparin, it has acquired considerable attention in the biomedical industry. In contrast, it also has other important properties, including anti-inflammatory, anti-viral, anti-thrombotic, and anti-tumor activity. Fucoidan has been used in recent years in the treatment of wound and burns.^51^ Anticoagulant activity of scaffolds fabricated with a combination of fucoidan with chitosan and polyvinyl alcohol (PVA) was studied by Zhang et al. Results showed that the fucoidan/chitosan/PVA scaffolds could be used with strong prospects for vascular tissue engineering, with great water absorption efficiency, ample porosity, decreased drug release and low cytotoxicity.^52^

### Silk sericin 

Silk sericin is a biocompatible protein component of *Bombyx mori*. In its structure, it includes amino groups and hydroxyl and carboxyl groups. It has an impact on wound dressings as it is well known to increase fibroblast development and the proliferation of human skin on cell density and collagen production on wound sites.^53^ This provides multiple advantages, including biocompatibility, toxicity, and biodegradability.

### Keratin

Keratin is a common biopolymer present in skin, nails and ears, wool plum, and vertebrate epithelia. The structure looks like a grid of 3D, which can be hydrogel.^54^ Keratin facilitates the healing process by engaging with the injury community of polyelectrolytes. Keratin can hold a lot of water in its form, making it a good choice for wound dressing as it can prevent the loss of biological fuels to the atmosphere and help reduce the exudation of injury.

## Inorganic Materials

Inorganic materials such as metallic nanoparticles and quantum dots have been used in diverse areas of biomedicine.

### Silver 

Mainly through the modification of the permeability of the bacterial membrane and the creation of reactive oxygen species (ROS), which destroy proteins, DNA, and other cellular components, silver nanoparticles may exert their non-bacterial behavior. The possible process is the release of silver ions from silver nanoparticles which create complex with thiol groups (-SH) of important enzymes, leading to inhibition of certain critical functions, such as cell division, DNA replication, and signal transduction.^55^ Silver nanoparticles are synthesized primarily by a chemical reduction of silver ions using reduction agents,^56^ borohydride, citrate, and ascorbate.

### Zinc

The antibacterial activity of zinc oxide nanoparticles is primarily due to their ROS generation property, similar to silver nanoparticles. Another mechanism suggested is the release of Zn^2+^ ions which harm bacteria through active inhibition of transport, amino acid metabolism, and disruption of enzymes systems.^57^

### Copper

The antibacterial activity of copper is related to the damage caused by the formation of a complex of thiol protein groups contributing to denaturation to the bacterial membrane. However, by inducing ROS development or attaching to DNA molecules, it can pursue an antibacterial behavior comparable to silver nanoparticles, and can interlink within and between nucleic acid strands to perturb its helical structure.^58^

## Methodologies to design nanofibers

### Electrospinning method

**Figure 2:**
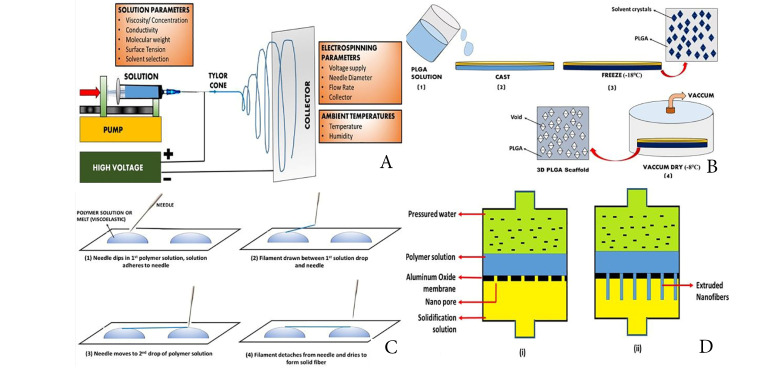
Figure showing techniques to develop nanofibers, (A) Electrospinning Technique; (B) Thermal-induced phase separation method; (C) Drawing Method; (D) Template Method for nanofiber

This method consists of a high-voltage source, a capillary tube with a small-diameter pipette or needles, and a metal screen. The polymer solution contains one element and the other is attached to the collector. At the end of the capillary tube, an electrical field is applied, contains the polymer solution held by surface tension and charges on the liquid surface.^59^ As the electric field is increased, a crucial value is reached at which repulsion electrostatic force overcomes the surface voltage and the activated fluid stream is expelled from the top of the Taylor cone. The released polymer solution jet is unstable and therefore elongates and lends the jet very long and thin. Then the loaded polymer fibres are solidified by evaporation of solvents.^60^ The collector is collected with randomly oriented nanofibers. In other collectors like the rotating drum, the metal frame^61^ and the two-parallel plates system, nanofibers can also be collected (Figure 2A). To produce nanofibers with uniform diameters and morphologies^62^ parameters such as jet stream movement and polymer concentration must be controlled. The nanofiber network of the electric spun is similar to the ECM.^63,64^

### Thermal-induced phase separation method

In this process, thermodynamic changes are used to distribute a homogenous polymer solution into a multi-phase system.^65^ The procedure involves 5 phases: polymer dissolution, separation of liquid-fluid or liquid-solid phase, polymer gels, water solvent extraction and freeze drying.^66^ This technique is used extensively to generate tissue regeneration scaffolds.^67^ This ensures the thermodynamically unstable solution of a homogenous polymer, leading to the separation of polymer-rich and polymer lean phases at appropriate temperatures. The polymer-rich phase solidifies to form the matrix following the removal of solvents, and the polymer-lean phase develops into pores. Next, the polymer solution can be divided into two types of phases according to the design: liquid fluids and solid fluids. Liquid-liquid separation is normally used to form bicontinuous phase structures while crystal structures are used for solid-liquid phase separation.^68^ The gelation step plays an important role in the control of the porous matrix morphology. The temperature, polymer, and solvent properties of gelation are influenced. The structure of a fibre network is regulated by temperature: low temperature of gelation results in the formation, while a platelet-like structure is generated by high gelation temperatures. Polymer levels affect the properties of the fibre: the increase in the concentration of polymer reduces porosity and increases mechanical characteristics, including tensile resistance. Properties of liquid influence scaffolds’ morphology.^69^ After gelation gel is placed for solvent exchange in distilled water (Figure 2B). The gel is subsequently removed from the water and freezes and freezes. It is then retained before characterization in a desiccator.^70^

### Drawing method

Long nanofibers can be produced one at a time in this method. The pulling process involves solidifying the dissolved spinning material in a solid fiber (Figure 2C).^65^ For melt spinning and solvent evaporation during dry spinning, a cooling phase is needed. However, this method has a limitation that this process can only produce nanofibers in a viscoelastic material that is extensively deformed while having sufficient cohesion to survive stresses developed during pulling.^71^

### Template synthesis 

This process is used to manufacture fibrils (solid nanofiber) and tubes (hollow nanofibers) use nanoporous membrane models, consisting of standardized pores of a cylindrical structure (Figure 2D).^72^ The fibrils and tubules of many material types are made by this method including metals, semiconductors, and electronically conductive polymers.^73^ Uniform pores allow fibres ‘ dimensions to be managed to create nanofibers with very small diameters by this process. Nevertheless, the downside of the process is that continuous nanofibers cannot be generated at once.^74^

### Self-assembly method

Such techniques are used to generate peptide nanofibers and peptide amphiphiles. During a self-assembly process, molecules are arranged in patterns or structures through a variety of driving forces, such as hydrophobic interactions, electrostatic forces, hydrogen bonding and van der Waals forces.^75^ Small polymeric molecules self- assembled through the formation of supramolecular hydrogels by poor interactions like hydrogen bonding and hydrophobic interactions. It is a successful technique for creating very thin nanofibers (less than 100 nm in diameter) of several micrometers in length.^76^

## Electrospun nanofibers for topical drug delivery systems 

There are various desired characteristics offered by electrospun nanofibers i.e. it is ideal for producing fibre mats using natural polymers like silk, ethylcellulose, chitosan, collagen, etc. and artificial polymers like poly (vinyl alcohol and poly (vinyl pyrrolidone).^77^ (ii) Fibre mats provide an effective distribution of hydrophilic as well as hydrophobic drugs with large surface-to-volume ratios.^78^ (iii) The specification for the product release can be tailored to the therapeutic application by modularizing several parameters, including drug to polymer interaction, diameter of the fibre, morphology and/or porosity.^79^ One way to modulate the release of drugs is by the processing of nanofibers (for example, polymerization in surface grafts).^80^ (iv) Electrospun fibre mats can provide sustained frequencies that improve patient adherence by reducing the frequency of topical application. (v) Electrospun fibre, with high interconnected porosity, can play an important role in mass transportation.^81^ (vi) Nanofiber meshes are malleable and suitable for the topical application of drug delivery. (vii) As part of a drug-releasing wound care system, fibre mats may be inserted into wound dressings.

## Mechanism of drug delivery from nanofibers

Nanofibers have the potential to deliver drugs at targeted site.^82^ Drug release kinetics are based on the porosity of nanofiber and the interaction between the drug matrix. Diffusion into surrounding tissues facilitated through the concentration gradient through which drugs are released. In cases where nanofibers include drug particles, gradual biodegradation of the nanofibers surface layers contributes to the release of the suspended material.^83^ This release mechanism can be associated with a burst release and correlated to the fast dissolution of surface particles.^42^ The embedded medication is secured by the shell-core technology.^43^ The current polymer’s enzymatic degradation is used to promote the release of the drug from nanofibers.^44^ Nanofibers may offer insight into specific penetration into scaffolds of the bioactive growth factors.^84^ Nanofibers in the ECM are considered to resemble collagen fibrils.

The multifold properties of nanofiber devices, namely high porosity, cell adherence efficiency, large surface area, cellular proliferation, and regulated *in vivo* drug release rate promote their candidacy for many biomedical applications including, scaffolds, drug delivery systems (DDSs), implants, prosthesis and wound care, tissue engineering, cancer diagnosis.^51,56^ It is also used in different fields, such as optical sensors, air filtration, oil-water isolation, textiles for sportswear, etc.

## Potential biomedical applications of nanofiber structures

### Regeneration of tissues

Greater surface area and similar structural features to the ECMs, nanofibers facilitate fast regeneration of tissues.^58^ Cell binding, prolongation, and adhesion were facilitated by nanofibers.^85^ The main function of nanofiber is to provide structural support as a replacement ECM until the normal ECM is regenerated. Regeneration of tissue is highly adaptive due to the use of biocompatible polymers and system optimization. Alginates, collagen, silk, and chitosan can regenerate the skin. A mixture of alginate, chitosan, chito-oligosaccharide and collagen (alginate-chitosan-cos-collagen) were utilized by Son et al for the development of various forms of hydrogels. Studies suggested that due to the porous structure of these scaffolds, it can be used as a replacement for skin tissue.^86^ In the diagnosis of many other kinds of tissues utilizing nanofibrous scaffolds, the promise has been shown in addition to the skin.^58^

### Novel drug delivery

Controlled delivery of proteins to targeted tissues can be facilitated through nanofibers. The nanofibers are used for embedding and therapy because their large surface area provides high therapeutic activity and reduces drug dissemination constraints which lead to an increase in the total amount of medicines released.^87^ When using nanofibers as a drug carrier, the drug must be immobilized in a polymer matrix to control the drug release mechanism.^88^ It is also necessary for nanofiber-based magnetic actuators to insert magnetic elements into the nanofiber membranes that can be helpfully transporting pharmaceutical items and clinical imaging.^59^ The adaptability of nanofibrous drug carriers makes it possible for this innovation to illustrate the promise for the treatment of various diseases.^61^

## Nanofibers drug delivery in different diseases ^89-109^

### Anticancer agents 

As the nanofibers exhibit excellent properties such as very high porosity, high surface area to volume ratio, and excellent physico-mechanical properties, they are widely used in biomedical applications (as shown in Table 2). Paclitaxel, water-insoluble anticancer drug can be delivered through surface modified mesoporous hollow stannic oxide nanofiber.

**Table 2:** Different studies related to nanofibers

** Drug**	**Category**	**Result**	**References**
Ofloxacin	Fluoroquinolone antibiotic	Article showed an accelerated effect on wound healing on superficial second degree burn in rats.	^89^
Silver sulfadiazine	Sulfonamides	The drug release was able to prevent the growth of a wide array of bacteria and accelerate the wound healing by preventing infection. Therefore it could accelerate the burn-wound closure rate.	^90^
Mupirocin	Antibiotic	The prepared PU/Mu composite scaffolds had satisfactory antibacterial activity especially against *Staphylococcus aureus*.	^91^
Acetaminophen	Analgesics and antipyretics	The developed NFs based layer can be applied either solely or in combination with other layers for desirable outcomes, for example, a multilayer dressing with this layer for burn pain management and other layer as antibiotics release for infection management.	^92^
Bromelain	Protein-digesting enzyme	Chitosan 2% w/v bromelain medicated textiles possesses great wound healing activity and could be considered as an effective natural topical burn wound healing treatment.	^93^
Gentamicin	Aminoglycoside antibiotic	The gentamicin released from the scaffolds showed a good inhibitory effect on the growth of bacteria and killed the microorganism. Thus, showing a great potential to be used as a wound healing material.	^94^
Mafenide Acetate	Sulfa antibiotic	Incorporation of mafenide acetate into chitosan/PVA nanofibers enhanced their antimicrobial activity against *Pseudomonas aeruginosa* and *S. aureus*.	^95^
Nitrofurazone	Nitrofuran class antibiotic	The prepared dual-layer nitrofurazone-loaded fiber dressings have satisfactory antibacterial activity against both gram-positive and gram-negative bacteria.	^96^
*Centella asiatica*, a medicinal herb	Effective against *S. aureus, Escherichia coli*, and *P. aeruginosa*	This article describes the diverse approaches that have been developed to produce electrospun nanofibres that are able to deliver naturally-derived chemical compounds in a controlled way and to prevent their degradation.	^97^
Silver acetate, silver tetrafluoroborate, silver nitrate, and silver phosphate	Antibacterial activity	The average diameters of the silver nanoparticles in gelatin nanofibers ranged between 13 and 25 nm, which was confirmed by transmission electron microscopy.	^98^
*Calendula officinalis*	Anti-inflammatory and antioedematous activities	This article indicated that *C. officinalis* extract is a suitable material for enhancing the biocompatibility of tissue engineering scaffolds.	^99^
Nisin	Antimicrobial drug	Novel nisin-loaded poly (vinyl alcohol)/wheat gluten/zirconia (Nisin-PVA/WG/ZrO_2_) nanofibrous membranes may have potential as a new nanofibrous membrane in drug delivery, wound dressing, and active food packaging.	^100^
*Calendula officinalis*	Anti-inflammatory and antioedematous activities	The results of *in vivo* experiments in rats suggested that HPGL– *C. officinalis* might be an interesting bioactive wound dressing material for clinical applications.	^101^
Ibuprofen	NSAIDs	Article demonstrated that scaffold properties are dependent on the environment in which they are placed and the importance of using serum, rather than saline, for initial in vitro evaluation of biofactor release from biodegradable scaffolds.	^102^
Silver sulfadiazine	Sulfonamides	Nanofibers with 0.6% silver sulfadiazine in zein, had shown excellent antibacterial activity for both gram-positive as well as gram-negative bacteria, so from this result we recommend 0.6% concentration of drug for further applications.	^103^
Asiaticoside	Antioxidant, anti-inflammatory, immunomodulatory	Its healing effect on deep partial-thickness burn injury of rats was obvious. Asiaticoside-loaded coaxial nanofibers provide a novel promising option for treatment of deep partial-thickness burn injury.	^104^
Silver sulfadiazine	Sulfonamides	The resultant nanofibers exhibited the appreciable antimicrobial activity against gram-negative *E. coli* and gram-positive *Bacillus subtilis* bacteria with considerable sustainability for repetitive use.	^105^
Mupirocin	Antibiotic	The fabricated nanofiber exhibited excellent hydrophilicity, cytocompatibility, sustained drug release, and antibacterial activity, which are favorable qualities for its use as a multifunctional material for wound dressing applications.	^106^
Ciprofloxacin	Antibiotic	Demonstrated promising wound resorption characteristics by using *in vivo* full-thickness excisional skin wound healing mice model.	^107^
Gabapentin and acetaminophen	Anticonvulsant and analgesics	The combination of quick release of strong nerve pain killer followed by slow release of mild pain killer reduced pain scores in a more professional manner with less side effects in burn patients.	^108^
Gentamicin	Antibiotic	The *in vitro* susceptibility tests confirmed that the gentamicin released from the liposomes immobilized at the surface of electrospun nanofiber matrix has bactericidal activity against *E. coli*, *P. aeruginosa* and *S. aureus*. The results showed that the developed system has promising performance for wound dressing applications, avoiding infections caused by these common pathogens.	^109^

### Antibiotics

Amoxicillin loaded nanofiber, Fluoroquinolone antibiotic (ciprofloxacin) loaded electrospun scaffold for ultrasound-assisted drug release, TiO_2_/AgNP-loaded cellulose acetate nanofiber scaffold, aminopenicillin loaded PEGylated poly(lactic-co-glycolic acid) electrospun nanofibers.

#### Anticancer agents Non-steroidal anti-inflammatory drugs (NSAIDs)

Polycaprolactone (PCL) nanofibers can enhance the dissolution rate of NSAIDs like ibuprofen and naproxen.^60^

### Cardiovascular agents

Nicorandil had been electrospun with polymeric nanofibers made out of riboflavin, hyaluronic acid, and PVA to set up a sublingual dose for treating angina pectoris, electrospun PCL nanofiber frameworks as a delivery carrier for the oral administration of poorly water-soluble drugs.

### Gastrointestinal Drugs

A core/shell nanofiber using PVA/PCL to load metoclopramide hydrochloride was fabricated.^54^

### Antihistamines

Incorporation of chlorpheniramine maleate into glutinous rice starch combining polyvinyl alcohol (GRS/PVA) electrospun nanofibers to research a drug shipping carrier idea and manipulate release houses of the nanofibers.^62^

### Contraceptives

Levonorgestrel loaded PVA nanofibers.

### DNA and RNA delivery

The nucleic acid in nanofiber scaffolds.

### Cosmetic applications

The versatility in topical methods has culminated in the use of more aware cosmetic products including medical goods and skin protection and regeneration products (such as facial masks, hair repair, and beauty therapy).

## Conclusion and future directions

Significant studies have been performed in production of nanofibers and many nanofiber the production technologies were developed because of its growing demand in industries. The most popular and usually used method for the manufacturing of nanofibers is electrospinning. Nanofibers are a technology with wide applications that have recently been developed. It can be adapted and used in different areas. It is costly to manufacture and therefore not favored on a large scale. Due to its intrinsic benefits together with mild processing conditions, extreme surface proximity to degree ratio and porosity, this increased importance of electrospinning in DDSs is attributable. In addition to those features, versatility, and simplicity of processing of nanofibers as properly as versions of the electrospinning machine which include coaxial electrospinning, emulsion electrospinning and aspect by side electrospinning in each the lab and at the commercial level gives electrospinning an advantage over historically utilized strategies which include section separation, spray drying unmarried emulsion and double emulsion cycle to fabricate drug-loaded nanofibers. While significant progress in DDS development has been achieved by electrospinning, an improvement is needed for the precise characterization of DDSs.

## Ethical Issues

There is no animal experimental carried out for this article.

## Conflict of Interest

The authors have no financial conflicts of interest.
